# Central Auditory Processing of Temporal and Spectral-Variance Cues in Cochlear Implant Listeners

**DOI:** 10.1371/journal.pone.0132423

**Published:** 2015-07-15

**Authors:** Carol Q. Pham, Peter Bremen, Weidong Shen, Shi-Ming Yang, John C. Middlebrooks, Fan-Gang Zeng, Myles Mc Laughlin

**Affiliations:** 1 Center for Hearing Research, University of California Irvine, Irvine, California, United States of America; 2 Department of Anatomy and Neurobiology, University of California Irvine, Irvine, California, United States of America; 3 Department of Otolaryngology- Head and Neck Surgery, University of California Irvine, Irvine, California, United States of America; 4 Institute of Otolaryngology, Chinese PLA Genera Hospital, Beijing, China; 5 Department of Neurobiology and Behavior, University of California Irvine, Irvine, California, United States of America; 6 Department of Biomedical Engineering, University of California Irvine, Irvine, California, United States of America; 7 Department of Cognitive Sciences, University of California Irvine, Irvine, California, United States of America; University of Salamanca- Institute for Neuroscience of Castille and Leon and Medical School, SPAIN

## Abstract

Cochlear implant (CI) listeners have difficulty understanding speech in complex listening environments. This deficit is thought to be largely due to peripheral encoding problems arising from current spread, which results in wide peripheral filters. In normal hearing (NH) listeners, central processing contributes to segregation of speech from competing sounds. We tested the hypothesis that basic central processing abilities are retained in post-lingually deaf CI listeners, but processing is hampered by degraded input from the periphery. In eight CI listeners, we measured auditory nerve compound action potentials to characterize peripheral filters. Then, we measured psychophysical detection thresholds in the presence of multi-electrode maskers placed either inside (peripheral masking) or outside (central masking) the peripheral filter. This was intended to distinguish peripheral from central contributions to signal detection. Introduction of temporal asynchrony between the signal and masker improved signal detection in both peripheral and central masking conditions for all CI listeners. Randomly varying components of the masker created spectral-variance cues, which seemed to benefit only two out of eight CI listeners. Contrastingly, the spectral-variance cues improved signal detection in all five NH listeners who listened to our CI simulation. Together these results indicate that widened peripheral filters significantly hamper central processing of spectral-variance cues but not of temporal cues in post-lingually deaf CI listeners. As indicated by two CI listeners in our study, however, post-lingually deaf CI listeners may retain some central processing abilities similar to NH listeners.

## Introduction

Cochlear implant (CI) listeners struggle to understand speech in complex environments whereas normal hearing (NH) listeners perform this task with apparent ease. In the latter group, sharp acoustic peripheral filters [1,2,3,4] allow for the resolution of individual harmonics [5] and permit separation of speech and interfering sounds into independent frequency channels [6]. The central auditory system can process strong spectral and temporal cues to group information from relevant frequency channels and form auditory objects in auditory scene analysis [7,8].

Compared to NH listeners, peripheral filters in CI users are much broader [9,10] because current spreads out along the cochlea and excites a large population of auditory nerve fibers [11,12]. This results in poor spectral resolution in the periphery and contributes to a CI user’s inability to separate speech from interfering sounds [13,14,15]. Electrical stimulation of the auditory nerve differs from acoustic stimulation in other notable ways. For example, acoustic stimulation generates stochastic firing patterns with phase-locking in the low frequency regions of the auditory nerve, whereas CI electrical-stimulation strategies cause entrainment in the nerve (i.e. action potentials strictly synchronized to the electrical pulses) up to rates of 800 Hz for stimuli 1–2 dB above threshold [16,17,18,19]. In addition to these differences in peripheral encoding, it is known that electrical stimulation causes neuroplastic changes in the central auditory system [20]. In spite of these central changes, we hypothesized that post-lingually deaf CI listeners may retain central processing abilities similar to NH listeners, but these abilities would be severely impaired by degraded peripheral encoding.

To test this hypothesis, we used multiple burst stimuli employed in standard informational masking paradigms [21,22] and customized them for CI listeners in a signal detection task. We used electrically evoked compound action potentials (ECAPs) to obtain a measure of peripheral filter bandwidth and then designed stimuli that elicited either predominantly peripheral or central (informational) masking. We evaluated central processing abilities by calculating the difference in detection thresholds, i.e. central masking release, between maskers with and without spectral variance (randomly varying masker components) and/or temporal asynchrony (onset delays) which are cues thought to be accessible to the central auditory system of NH listeners [21].

We conducted two experiments using these stimuli. Experiment I showed that CI listeners could use the temporal cues to gain release from central masking, indicating that central processing of temporal cues by CI listeners was similar to that in NH listeners. Unlike NH listeners, however, most CI listeners could not exploit the spectral-variance cues to gain central masking release. In Experiment II we simulated implant listening in NH listeners and showed that wide peripheral filters degraded the spectral-variance cues leaving the temporal cues intact. Furthermore, large inter-listener variability amongst both NH and CI listeners suggested intrinsic differences in central processing capabilities, which may affect sound segregation with degraded peripheral input.

In summary, all results indicated that central processing of the temporal cues in post-lingually deaf CI listeners is similar to that in NH listeners and is largely unaffected by peripheral encoding differences. Degraded peripheral encoding in CI listeners, however, likely limits the use of the spectral-variance cues by the central auditory system under electric stimulation.

## General Methods

### Ethical statement

The University of California Irvine’s Institutional Review Board approved all experimental procedures for both CI and NH listeners. Written informed consent was obtained from each listener and listeners were compensated for their participation on an hourly basis.

### General experimental setup and procedure

All testing with CI and NH listeners was performed in a double-walled sound-attenuating booth.

The initial step was to measure threshold and comfort levels in all CI listeners to assess their dynamic ranges. To map comfort level, CI listeners judged signal loudness based on a standard 10-interval loudness scale (0 = no sound, 1 = barely audible, 6 = most comfortable, 10 = extremely loud). We asked CI listeners to indicate when they perceived the given stimulus to be most comfortable (6/10). Comfort level represented a conservative measure of loudness to avoid overstimulation with multi-component stimuli. We used a standard 3-down-1-up, two-alternative force choice (2AFC) paradigm [23] to track thresholds of unmasked signals (threshold level of CI listeners) and calculated the dynamic range as the difference between comfort and threshold level per intracochlear electrode.

We used the same 2AFC procedure to measure psychophysical detection thresholds of masked signals in Experiments I (CI listeners) and II (NH listeners). For CI listeners, we set the starting level to the listener’s comfort level. To accelerate threshold convergence, we set the signal level in consecutive repetitions to 40% percent of the dynamic range of the previously tracked threshold with the provision that the starting level could never exceed the comfort level. The subject listened to two intervals indicated by buttons labeled ‘1’ and ‘2’ on a graphical user interface (MATLAB, The Mathworks, Natick, MA). The buttons sequentially illuminated as the stimuli played. By random selection, one interval contained the signal and masker while the other interval contained the masker alone. We instructed listeners to select the interval that contained the signal via mouse click. As visual feedback after each trial, the interval button turned green or red to indicate a correct or an incorrect response, respectively. We calculated signal detection threshold as the average over the last six out of ten reversals.

### Statistics and data analysis

Absolute signal detection thresholds across listeners were not normally distributed. Therefore, a non-parametric, repeated measures, Friedman’s test was used to compare masker conditions. A statistical significance level of *p* < 0.05 was used with *post-hoc* Bonferroni adjustments. We used linear regression with a least-squares criterion to assess correlations between masker conditions and a two-sample Kolmogorov-Smirnov test to determine significant differences (p < 0.05) between paired conditions. All statistical analysis was performed using MATLAB.

## Experiment I: Signal Detection with Peripheral and Central Maskers

### Rationale

A number of studies in NH listeners have used non-speech, multi-tone maskers with a protected band centered at the signal frequency [21,24,25,26,27] to study the effects of energetic and informational masking. Energetic masking is defined as masking due to the excitation of overlapping neural populations. Although the term informational masking remains elusive, it is traditionally defined negatively as any masking that is non-energetic in origin [28]. In CI listeners, peripheral filters (i.e. a measure of cochlear spread of neural excitation) can be measured using ECAPs [29,30,31]. In the present study, we used this filter to define the terms ‘peripheral’ and ‘central’ masking which are parallel to, but not strictly synonymous with, the terms ‘energetic’ and ‘informational’ masking. Consequently, we define peripheral masking as an increase in detection threshold of a signal electrode due to the presence of masking electrodes located within the peripheral filter of the signal. Furthermore, we define central masking as an increase in detection threshold of a signal electrode due to stimulation of masking electrodes located outside the same peripheral filter.

Results from previous studies [21,24,25,26,27] indicated that NH listeners can experience large amounts of central masking and that manipulation of the temporal and spectral content of the stimuli can facilitate central masking release (i.e., decrease signal detection thresholds using stimulus cues). Experiment I had three goals: 1) to determine if CI listeners experience similar amounts of central masking as NH listeners; 2) to determine if CI listeners could access similar temporal and spectral-variance cues to gain release from central masking; and 3) to identify peripheral encoding mechanisms which may impair central processing.

### Experiment I. Methods

#### Cochlear Implant listeners

We screened 22 implants in fifteen CI listeners, including 7 bilateral implant listeners, with Nucleus 24, Nucleus 5, or Freedom devices (Cochlear Corporation, Australia) to meet three inclusion criteria. First, CI listeners needed to have a dynamic range greater than 20 clinical units to enable testing of different masker levels. Second, CI listeners needed to have a measurable ECAP filter on electrode 11 (signal electrode) that could be well fit by a Gaussian function (R^2^ ≥ 0.80) with a standard deviation ≤±7 electrodes so that multi-electrode maskers could be placed either inside or outside the peripheral filter. Third, our sample of CI listeners had to have 22 active electrodes switched on to enable testing of different masker electrode configurations. Out of the fifteen listeners screened, two listeners failed the first criterion, three listeners failed the second criterion (two listeners had filter widths >~±8 on electrode 11; one listener did not have measurable ECAPs) and two listeners failed the third criterion. Eight post-lingually deaf, adult listeners met all three criteria (five females; age 51–85; mean age 70). Table 1 provides additional demographic and audiological details for these eight listeners.

**Table 1:** CI listeners’ demographic and audiological details.

Listener	Sex	Age	HL age	Deafness duration (y)	Etiology	HA (y)	CI (y)	Ear tested	CI device (no. of CI)	DR signal E11 (μA)
CI 1	F	70	45	25	Presbycusis	53	3	R	Freedom (1)	435
CI 2	F	75	30	25	Hereditary	33	8	L	N24 (2)	183
CI 3	M	78	63	7	Acoustic trauma	45	6	R	N24 (2)	148
CI 4	F	74	35	16	Hereditary	35	2	R	N24 (2)	187
CI 5	M	85	58	23	Hereditary	60	3	R	N24 (1)	210
CI 6	F	51	18	32	Hereditary otosclerosis	20	2	R	Freedom (2)	296
CI 7	M	53	15	38	Autoimmune disease	23	2	L	N5 (2)	185
CI 8	F	70	15	50	Ototoxicity	39	8	L	Freedom (2)	225

Age, refers to age during experimental testing; HL age, refers to age of hearing loss onset; deafness duration, refers to number of years since the onset of profound hearing loss; HA, refers to number of years of hearing aid usage in the tested ear; CI, refers to number of years of cochlear implant usage in the tested ear; CI device (number of CI), Freedom means a Nucleus Freedom device, N24 and N5 means a Nucleus 24 and Nucleus 5 device, respectively; DR signal E11 (μA), refers to dynamic range of stimulus signal presented on electrode 11 in microamperes.

#### Peripheral filter measurement using Electrically Evoked Compound Action Potentials

**Fig 1:**
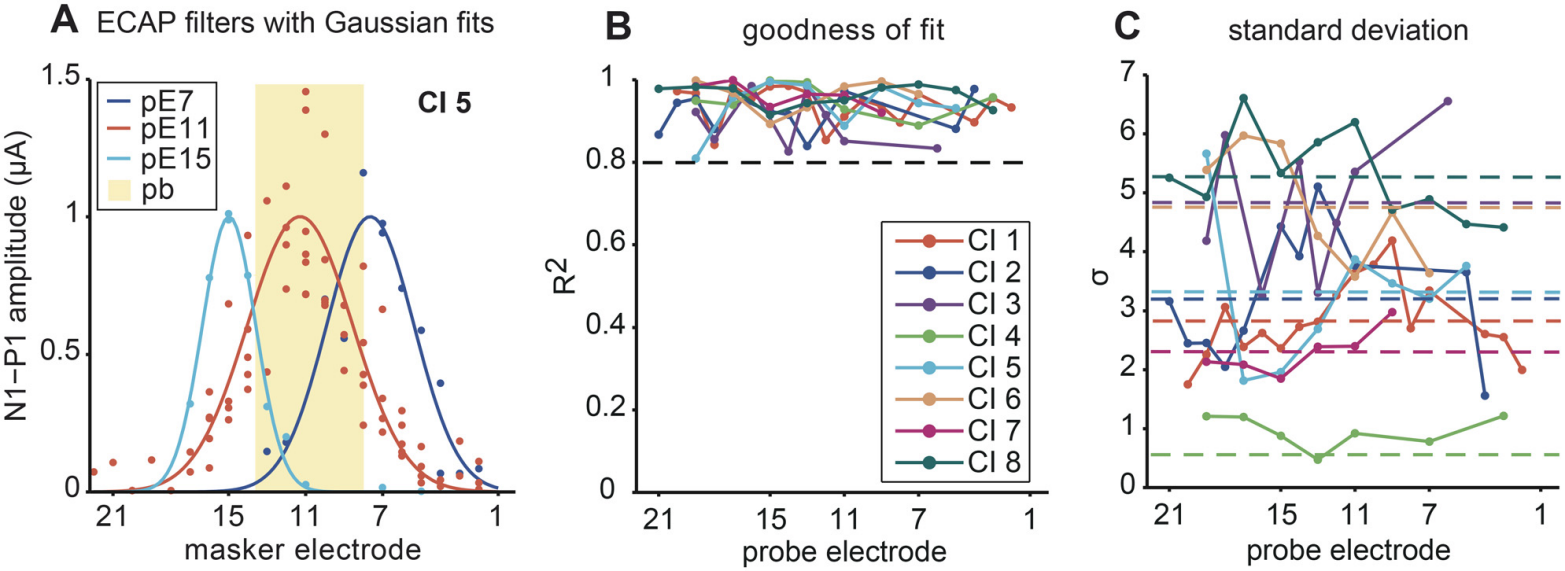
Characterization of CI peripheral filters. (A) Electrically evoked compound action potential (ECAP) filters with Gaussian fits. Spread of excitation profiles are shown for three probe electrodes (pE7, pE11, pE15) in listener CI 5. ECAP N1-P1 amplitudes and Gaussian fits are indicated with circles and lines, respectively. (B) Goodness of fit. R^2^ between the measured filter and the Gaussian function for each tested probe electrode in all CI listeners (different colors) are shown. The black dashed line demarcates where fits are equivalent to or higher than R^2^ = 0.8. (C) Standard deviation. Peripheral filter bandwidth (σ) was estimated as a function of probe electrode. Each colored, dashed line corresponds to the average standard deviation across probes per CI listener. Based on these data we estimated a protected band [pb; shaded region in (A)] of ±3 electrodes to best separate peripheral and central masking effects (see text for details).

We used Custom Sound EP (Cochlear Corporation, Australia) software to record ECAPs in the CI listeners. We employed the forward masking protocol previously described by Brown and colleagues [29], which uses a subtraction technique in a masker-probe paradigm to separate the ECAP from the stimulation artifact. To capture the spread of excitation along the cochlea, we moved the masker across the electrode array while we fixed the probe and recording electrodes. Extracting the N1-P1 amplitude of separate ECAP responses measured at each masker electrode location gave a measurement of one peripheral filter. The protocol used charge-balanced, biphasic pulses delivered in monopolar stimulation mode through the listener’s implant. Recording parameters were optimized for eliciting neural responses per CI listener. We used typically a pulse duration of 25 μs/phase, an interphase gap of 7 μs and a pulse rate of 40 (probe) and 100 (masker) pulses/s and set the delay between masker and probe pulse to 400 μs. Both masker and probe pulse amplitude were set to the listener’s most comfortable loudness level. We used the extracochlear electrode MP1 as the reference electrode and an intracochlear electrode as the recording electrode, the latter offset by two positions from the probe electrode. The delay between probe pulse and recording buffer was on average 100 μs and we set the amplifier gain to either 40 or 50 dB. The Custom Sound EP software automatically extracted N1-P1 ECAP amplitudes [31], which we inspected visually and corrected manually when necessary. A characterization of CI peripheral filters is shown in Fig 1 including examples of filter fits for three probes tested in one CI listener CI 5.

#### Quantifying peripheral ECAP filters

To quantify peripheral filters, we exported ECAP N1-P1 amplitudes from Custom Sound EP and fitted them with a Gaussian curve using the lsqcurvefit function in MATLAB. The following formula describes the Gaussian fit,

$$I\;=\;10^{}\mu A*175^{CU/255}$$ (1)

where *a* is the amplitude of the filter, *x* is the masker electrode number, *μ* is the mean (i.e. probe electrode), and *σ* is the standard deviation (i.e. bandwidth of the ECAP filter). To facilitate comparison between filters of an individual listener, we normalized them by dividing by the maximum amplitude of a given filter fit (Fig 1).

#### Electrical stimulation and electrode mapping for perceptual studies

We presented all electrical stimuli using a research interface (HEINRI) [32]. In four bilateral CI listeners (CI 4,6,7,8) we stimulated the second implanted ear and in the two other bilateral cases (CI 2,3) we stimulated the first implanted ear after measuring ECAPs in both ears. We tested the second implanted ear for the following reasons: the electrode array was not fully inserted in the first implanted ear (CI 4); the first implant had inactive electrodes (CI 6, CI 8); the second CI had sharper ECAP filters (CI 7). We set the extracochlear electrodes, MP1 and MP2, as return electrodes and stimulated in monopolar mode. The pulse width, interphase gap and pulse rate remained fixed for all stimuli at 25 μs/phase with a 10-μs interphase gap and a pulse rate of 300 pps per channel. When multiple electrodes were stimulated we used continuous interleaved sampling with the pulse on the most basal electrode occurring first, and the following pulse on the next electrode in the apical direction, occurring 125 μs after the onset of the previous pulse.

Using a signal matched to that used with the peripheral and central masking stimuli (see below), we mapped comfort and threshold levels on all electrodes. The signal consisted of four bursts of a 40-ms pulse train presented on one electrode at a rate of 5/s. In the first step, we decreased stimulation level by 5 clinical units, 3 clinical units in the two subsequent steps and 1 clinical unit increments thereafter. We determined the threshold level as the average of the last six out of ten reversals. A map of dynamic range was created and used for subsequent testing. We checked these maps periodically, but did not observe significant shifts during the testing period.

The HEINRI system allows specification of pulse amplitude levels in clinical units (CU; range 0 to 255 in steps of 1) which is related to current (I) by the following formula,

$$I\;=\;10^{}\mu A*175^{CU/255}$$ (2)

During all threshold tracking runs, current levels were controlled, adjusted and stored in CU. In the results section, we report tracked thresholds in units of current (μA) derived from Eq 2.

#### Peripheral and central masking stimuli

**Fig 2:**
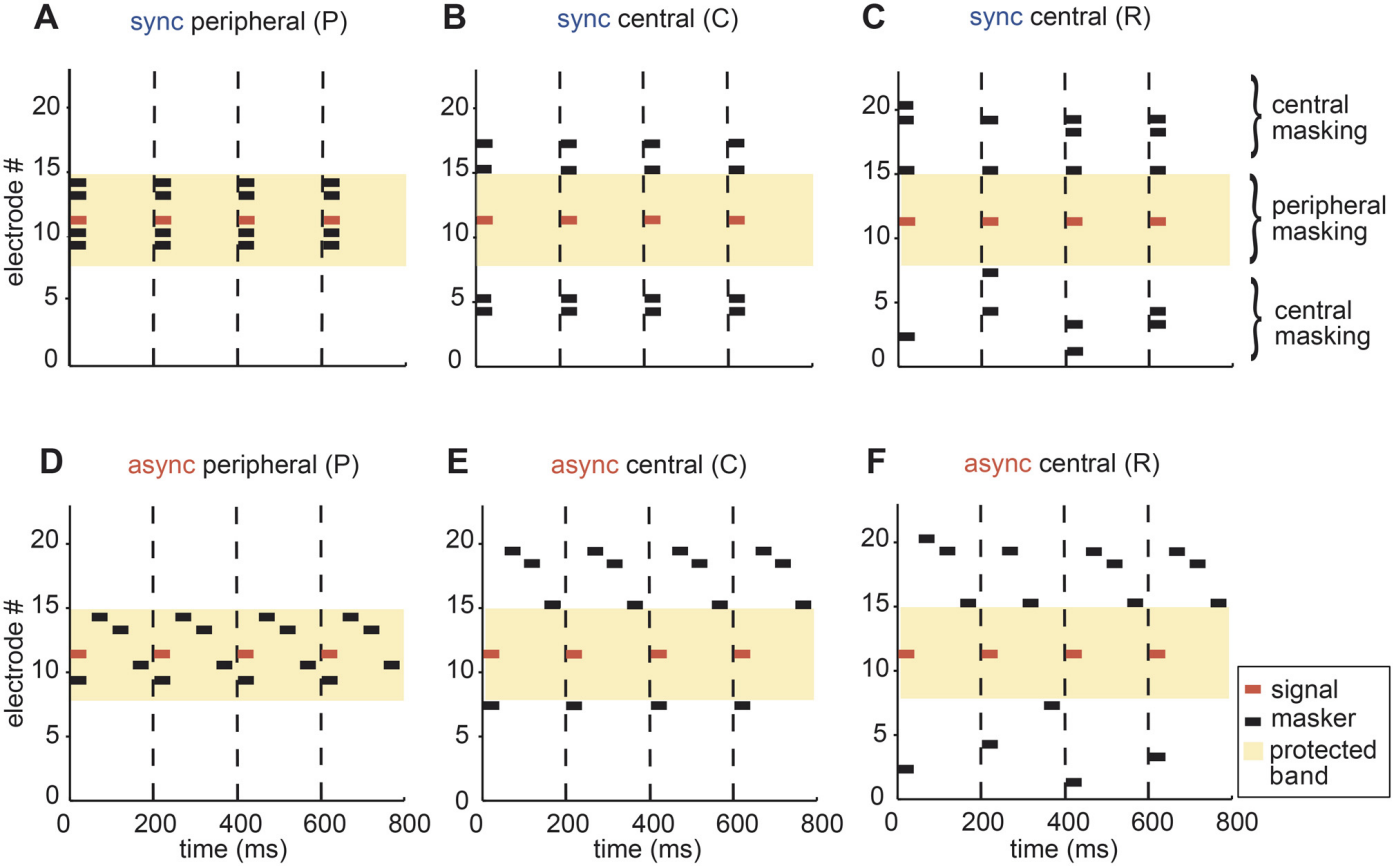
Peripheral and central masking stimuli tested in CI listeners. Multi-electrode maskers (black) were placed either inside [peripheral masking; P masker: (A), (D)] or outside [central masking: (B), (C), (E), (F)] the peripheral filter (marked by the protected band, shaded region). The signal (red) was fixed on electrode 11. Four randomly chosen masker electrode positions outside of the filter remained constant [C masker: (B), (E)] or randomly varied [R masker: (C), (F)] between bursts over time. Maskers were presented in two temporal conditions. In the synchronous condition [sync maskers: (A-C)] masker components gated on synchronously *re* signal and in the asynchronous condition [async maskers: (D-F)] the four independent masker components had onset delays of 0, 50, 100, and 150 ms *re* signal (see text for details).

We designed stimuli that intended to separate peripheral and central masking effects. Based on the ECAP peripheral filter measurements, we estimated filter bandwidths as the standard deviation (σ) of the Gaussian fit (shaded protected band (pb), Fig 1A). In all experiments, the signal was presented on electrode 11. Both the signal and masker were 40-ms bursts pulsed at a rate of 5/s. Maskers comprised of four electrode components per burst. Masker components could either be within (peripheral masker, Fig 2A and 2D) or outside (central masker, Fig 2B, 2C, 2E, and 2F) the ECAP filter.

To study central masking release based on spectral variance across masker bursts (i.e. spectral-variance release), we compared conditions in which the four masker electrode positions remained constant between bursts (C masker, Fig 2B and 2E) with conditions in which the four masker electrode positions varied randomly between bursts (R masker, Fig 2C and 2F) over time. In NH listeners the R-like stimulus yields lower signal detection thresholds when compared to C-like stimulus [21,27]. Peripheral (P) masker components remained constant between bursts due to the restricted number of distinct electrodes within the protected band.

Additionally, we examined masking releasing due to a temporal cue (i.e. temporal release) by presenting maskers both synchronously and asynchronously *re* signal in separate conditions. Note that the delay between biphasic pulses on different electrodes was 125 μs, leading to a slight temporal offset among bursts on various electrodes even in the nominally synchronous condition, but this delay was negligible compared with delays on the order of milliseconds between bursts in the asynchronous condition. In the synchronous conditions, the onset of the four masker bursts and the signal was synchronous (given the technical limitations of the stimulation device) (Fig 2A, 2B and 2C). In the asynchronous conditions, each masker burst had a random onset delay of 0, 50, 100 and 150 ms *re* signal (Fig 2D, 2E and 2F). Thus, in asynchronous conditions, one masker component overlapped with the signal and the other three components were separated in time from the signal and from each other. This yielded a total of six masking stimuli: peripheral (P) and central (C, R) maskers in synchronous (sync) and asynchronous (async) timing conditions (this convention for labeling the stimulus condition will be used henceforth). We selected stimulus parameter settings to closely match those previously tested in NH listeners [27] for the purpose of comparing masked signal detection in electric vs. acoustic hearing.

#### Using ECAP filters to separate peripheral and central masking effects

We used filters derived from ECAP measurements to determine the degree to which each electrode contributed to either peripheral or central masking. ECAPs were measured with the masker and probe electrode fixed at the most comfortable level on the test electrode. During threshold tracking experiments, the masker electrodes were fixed at 10%, 30% or 50% DR (dynamic range of each masking component) while the signal electrode started at comfort level and was generally tracked at a threshold below this level. Note that as the current level decreases so does the spread of neural excitation, meaning that the excitation profiles elicited during threshold tracking experiments will be narrower than those measured in the ECAP experiment. Thus, for central masking stimuli, it is reasonable to assume that masking electrodes outside the protected band excite a different auditory nerve fiber population than that responding to the signal electrode inside the protected band. For the peripheral masking stimuli, however, it is likely that electrodes placed inside the protected band stimulate a nerve fiber population that mostly, but not completely, overlaps with the population excited by the signal electrode. This means that the peripheral masking stimuli can potentially create some limited central masking effects. A limitation of relating ECAPs to behavioral masking may be that response amplitudes do not exclusively represent the spread of excitation. Although the amplitude of the ECAP can approximate the number of neurons responsive to a specific stimulus, this method assumes that the recording electrode primarily measures the response from neurons near that electrode without full determination of the degree of spatial filtering. Consequently, the response amplitude as a function of recording positioning for a fixed level and position of stimulating electrode depends on the spread of excitation across fibers and the spread of the response fields from each active neuron to the recording electrode [33].

#### 2AFC paradigm for testing CI listeners

For all tests we held the masker level constant at a fixed percentage of the dynamic range and adjusted the signal level using the 2AFC threshold tracking procedure. We used a protected band of ±3, ±5, or ±7 electrodes centered at the signal electrode and measured detection thresholds at three different masker levels (10%, 30% and 50% DR; only 30% DR was tested with different protected bands).

Like in the initial mapping of threshold level, the signal level decreased by 5, 3, 1 clinical units for the first, second and third, and the subsequent reversals, respectively. We repeated threshold measurements five times per listener. Each interval within a trial contained maskers with freshly drawn electrode components. We presented all six stimuli randomly during a session (3–5 repetitions per masker condition lasting about 240–300 mins with at least 10-min breaks after every 30 mins of testing) and held the masker condition constant during one threshold measurement (~100 trials lasting about 5 mins). Each of the six maskers was tested before any condition was repeated. We roved the level of the masker-only interval by ±3 clinical units to minimize the contribution of level cues between the masker-only and the signal-plus-masker intervals (four vs. five electrodes).

#### Training

To minimize learning effects, all listeners underwent two types of training sessions prior to data collection. In the first training session, listeners used a graphical user interface to listen to the signal alone, all six maskers alone, and each signal-plus-masker condition to become familiar with the stimuli. The second training session used the threshold tracking paradigm outlined above with the masker level set to 10% of the dynamic range. We trained listeners using the asynchronous R masker, generally the easiest masker condition, to help listeners become accustomed to the test procedure.

#### Quantifying spectral-variance release from central masking

We calculated the amount of spectral-variance release from central masking by subtracting median R from median C thresholds. In order to assess the amount of variability, we used a bootstrapping method. First, we randomly drew three repetitions out of the total five threshold measurements with replacement and calculated the mean over these repetitions. We repeated this procedure 1000 times and finally calculated the standard deviation over these bootstrapped means. We also applied this analysis to NH data from Experiment II.

### Experiment I. Results

#### ECAP spread of excitation

Fig 1A shows the ECAP filters in one listener (CI 5) with the N1-P1 amplitudes (circles) and corresponding Gaussian fits (lines). This figure shows filters measured with the probe electrode (pE) at three different locations (electrode 7, 11 and 15). Fig 1B summarizes the goodness of fit between the measured filters and the Gaussian fit (Eq 1) for each of the eight CI listeners who participated in the study. In all listeners, the spread of excitation profiles were measured for a range of different probe electrodes spanning the length of the electrode array.

Gaussian fits for 80 out of 103 probe electrodes yielded R^2^ ≥ 0.8 (Fig 1B) whereas the other 23 probes were edge electrodes yielding weak or no neural responses. Initial visual inspection suggested that a Gaussian model would be a reasonable approximation of individual's filter shape and high R^2^ values justified use of this simple model. For the purpose of estimating bandwidth for stimulus generation (criteria for designating electrode positions inside vs. outside the peripheral filter), the Gaussian model provided the best approximation of filter shape without resorting to complicated multi-parameter models.

Even after careful subject selection, fitted ECAP filter bandwidth (standard deviation; σ in Eq 1) varied largely across listeners and electrodes, ranging from 1–7 electrodes wide (Fig 1C, colored dashed lines). Listener CI 4 had the narrowest filters with standard deviations close to 1, whereas listener CI 8 tended to have the widest filters. The three different protected bandwidths selected for testing (±3, ±5 and ±7 electrodes) helped to accommodate for some of this variability. The influence of the protected bandwidth on central and peripheral masking is further explained below and is illustrated here with the following examples: At one extreme in listener CI 8, a protected band of ±3 electrodes may not have been wide enough to ensure that central masking stimuli did not create some unwanted peripheral masking. In this listener, a protected band of ±7 electrodes would have minimized peripheral masking effects with the central masking stimuli. At the other extreme in listener CI 4, a protected band of ±3 electrodes may have meant that the peripheral masking stimuli created some unwanted central masking effects. Because the peripheral masking stimuli required a bandwidth of at least five electrodes (one signal plus four masker electrodes) a narrower protected band was not tested. Thus, the protected band may not have always completely distinguished central and peripheral masking effects in all listeners. It did, however, always limit any unwanted peripheral masking effects when central masking stimuli were presented and vice-versa.

#### Signal detection with peripheral and central masker

**Fig 3:**
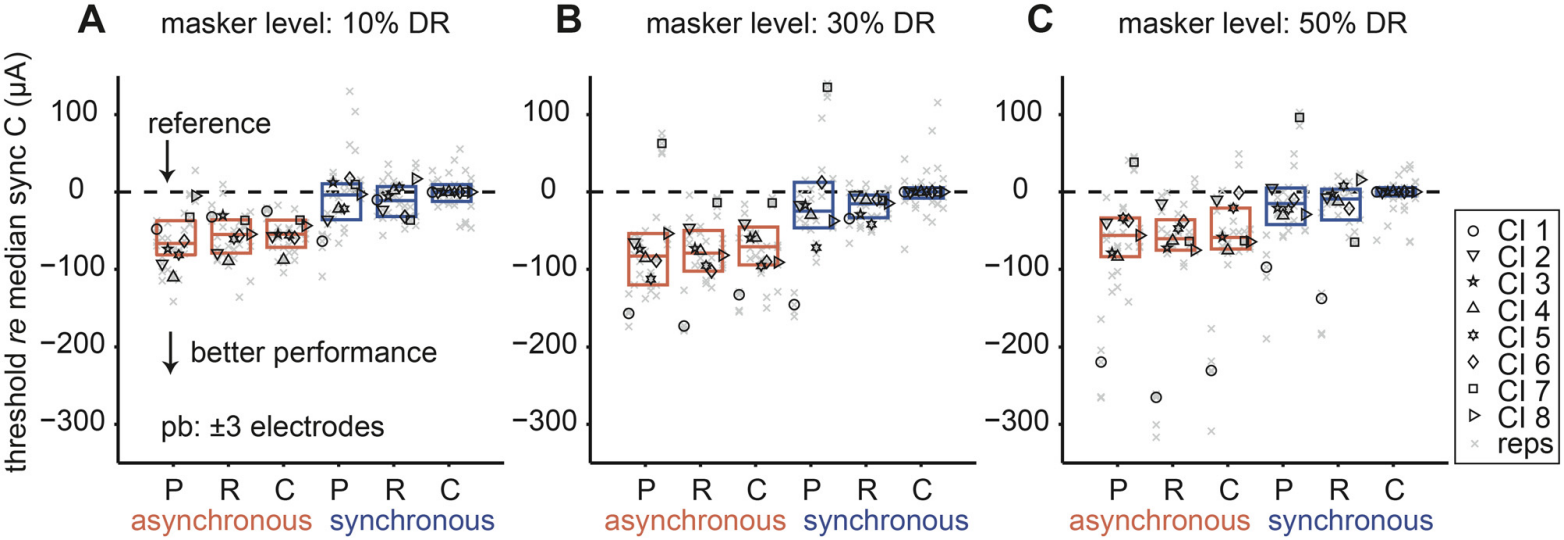
Effect of masker level on signal detection thresholds. Capital letters on abscissa correspond to peripheral (P) and central (C, R) maskers in both asynchronous (red) and synchronous (blue) timing conditions. Signal detection thresholds (*re* each CI listener’s median, synchronous C threshold) are plotted as a function of stimulus. Detection thresholds are expressed in current (μA) and were tracked at three masker levels expressed as a percentage of dynamic range: (A) 10%, (B) 30% and (C) 50% DR. The protected band (pb) was set to ±3 electrodes. Different symbols indicate individual listeners’ median thresholds and gray x’s indicate individual repetitions. The dashed line marks the reference point. The mid-line of the boxes indicate the group median and the lower and upper lines indicate the 25^th^ and 75^th^ quartiles, respectively. Lower values indicate less masking (i.e., better performance).

Fig 3 presents the results from the six stimulus conditions at three masker levels (10%, 30% and 50% DR shown in A-C, respectively) using a protected band (pb) of ±3 electrodes. Each listener’s median detection threshold for a specific condition, hereafter referred to as a detection threshold, is shown as a different symbol. Individual repetitions are shown as gray x’s. The midline of the boxes indicates the group median and the lower and upper lines indicate the 25^th^ and 75^th^ quartiles, respectively (red and blue indicate asynchronous and synchronous conditions, respectively). Absolute comfort and threshold levels varied widely across CI listeners resulting in greatly varying absolute detection thresholds (not shown). For that reason, each threshold was referenced to the listener’s detection threshold in the synchronous C condition per masker level (dashed lines, Fig 3). As a result, median synchronous C thresholds per CI listener were plotted as zero in each plot with negative detection thresholds indicating masking release (i.e. better performance) relative to this generally most difficult condition.

#### Temporal release

**Fig 4:**
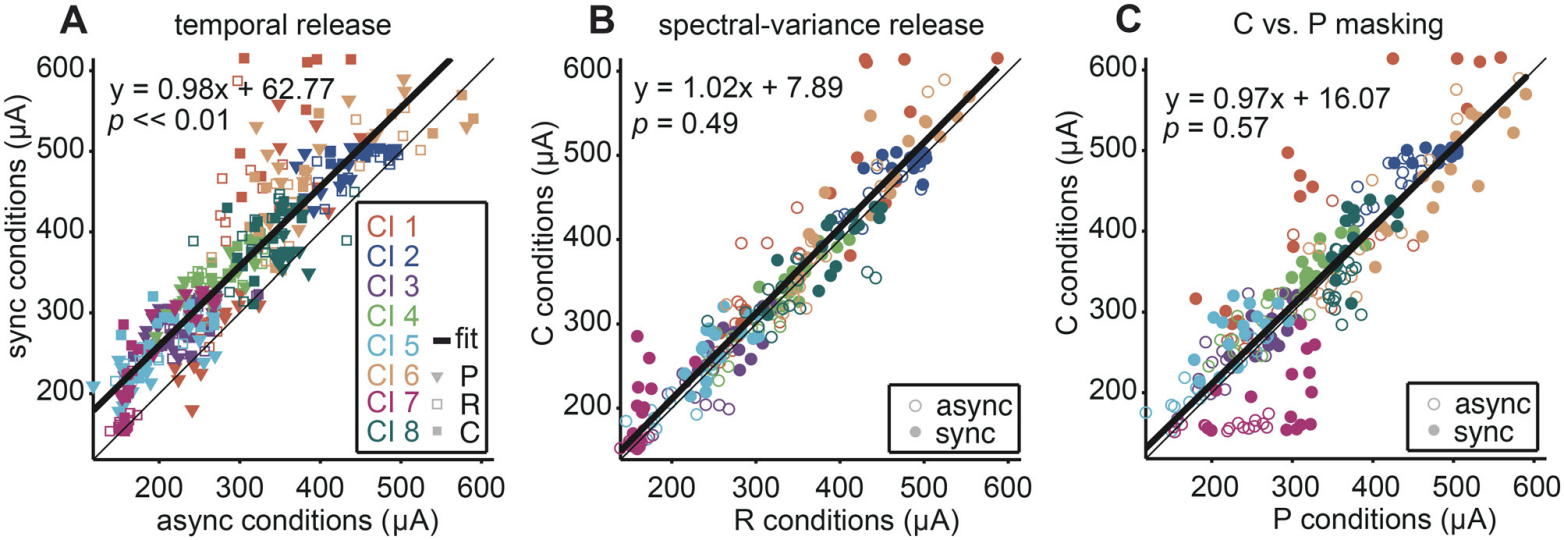
Correlation analysis quantifying temporal and spectral-variance release. In all plots, individual listeners are indicated by different colors. Data from all masker levels with a protected band of ±3 electrodes are plotted. Repetitions were paired within the same masker level and timing condition. The unity line and a linear fit through the data are shown as thin and thick lines, respectively. Each plot shows the equation describing the linear fit and the *p* value from the Kolmogorov-Smirnov test. All linear fits were significant, *p* > 10^5^, with R^2^ = 0.90, 0.96, and 0.89 in (A), (B), and (C) respectively. (A) Temporal release. Absolute detection thresholds with synchronous (sync) maskers are shown as a function of thresholds with asynchronous (async) maskers. Inverted triangles represent peripheral (P) maskers whereas open and filled squares represent central (C, R) maskers, respectively. (B) Spectral-variance release. Absolute detection thresholds with C maskers are shown as a function of thresholds with R maskers. Open and filled circles represent async and sync timing conditions, respectively. (C) Central vs. Peripheral masking. Absolute detection thresholds with C maskers are shown as a function of thresholds with P maskers. Open and filled circles represent async and sync timing conditions, respectively.

Gating the maskers on asynchronously introduced a temporal cue in the stimuli that could potentially facilitate signal detection. It was clear across all masker levels that the asynchronous condition produced lower detection thresholds than the synchronous condition (Fig 3). A Friedman’s test with correction for multiple comparisons showed that this effect was significant (*p* < 0.01). A correlation analysis further quantified this effect for thresholds in both timing conditions. We plotted individual absolute thresholds from synchronous conditions as a function of the corresponding asynchronous conditions with paired repetitions within the same masker level condition and timing condition (Fig 4A). Ninety-two percent of the points fell above the unity line, meaning that the asynchronous condition yielded significantly lower detection thresholds than the synchronous condition (Kolmogorov-Smirnov test, *p* << 0.01). All listeners experienced greater masking with synchronous than asynchronous maskers (>90% of points above the unity line). A linear regression (*R* ^2^ = 0.90; *p <* 10^−5^) yielded an offset of ~63 μA between synchronous over asynchronous timing conditions and further support that temporal cues facilitate signal detection. The effect was most pronounced in listener CI 1 (red). Furthermore, this analysis suggested that a temporal cue could facilitate release from both peripheral and central masking (Fig 3). The finding that temporal cues could facilitate signal detection in complex listening situations is in agreement with reports using similar stimuli in NH listeners [e.g., 27] as well as auditory scene analysis studies in CI listeners [34,35].

#### Spectral-variance release

In NH listeners, spectral variance in addition to a temporal cue can facilitate central masking release [21,27,36]. Spectral variance in the present experiment was introduced by randomly varying electrode positions (frequencies) in the central masker across bursts (R masker) while the signal remained constant across bursts. Across masker level and timing conditions, there was a weak non-significant tendency for the R thresholds to be lower than the C thresholds (Fig 3) (*p* > 0.05; Friedman’s test using the Bonferroni correction for multiple comparisons).

Fig 4B displays thresholds for all central (C and R) conditions in the same scatter plot format as used previously. Sixty-five percent of the points fell above the unity line, indicating that C and R detection thresholds were similar, but that there was a weak tendency for C thresholds to be higher than R thresholds with large individual differences ranging from 47%-83% of points above the unity line. A linear fit to the data showed a small offset of ~8 μA between the central masking conditions (*R* ^2^ = 0.96; *p <* 10^−5^). In general, spectral variance did not facilitate central masking release in most CI listeners (Kolmogorov-Smirnov test, *p* = 0.49). In Fig 3, listeners CI 1 (circle), CI 7 (square), and to a lesser degree CI 6 (diamond) were notable exceptions in that their detection thresholds were similar to those seen in NH listeners with lower R thresholds relative to P and C thresholds at all masker levels. For listener CI 6, this pattern existed only at 10% masker level. These results failed to reach significance.

**Fig 5:**
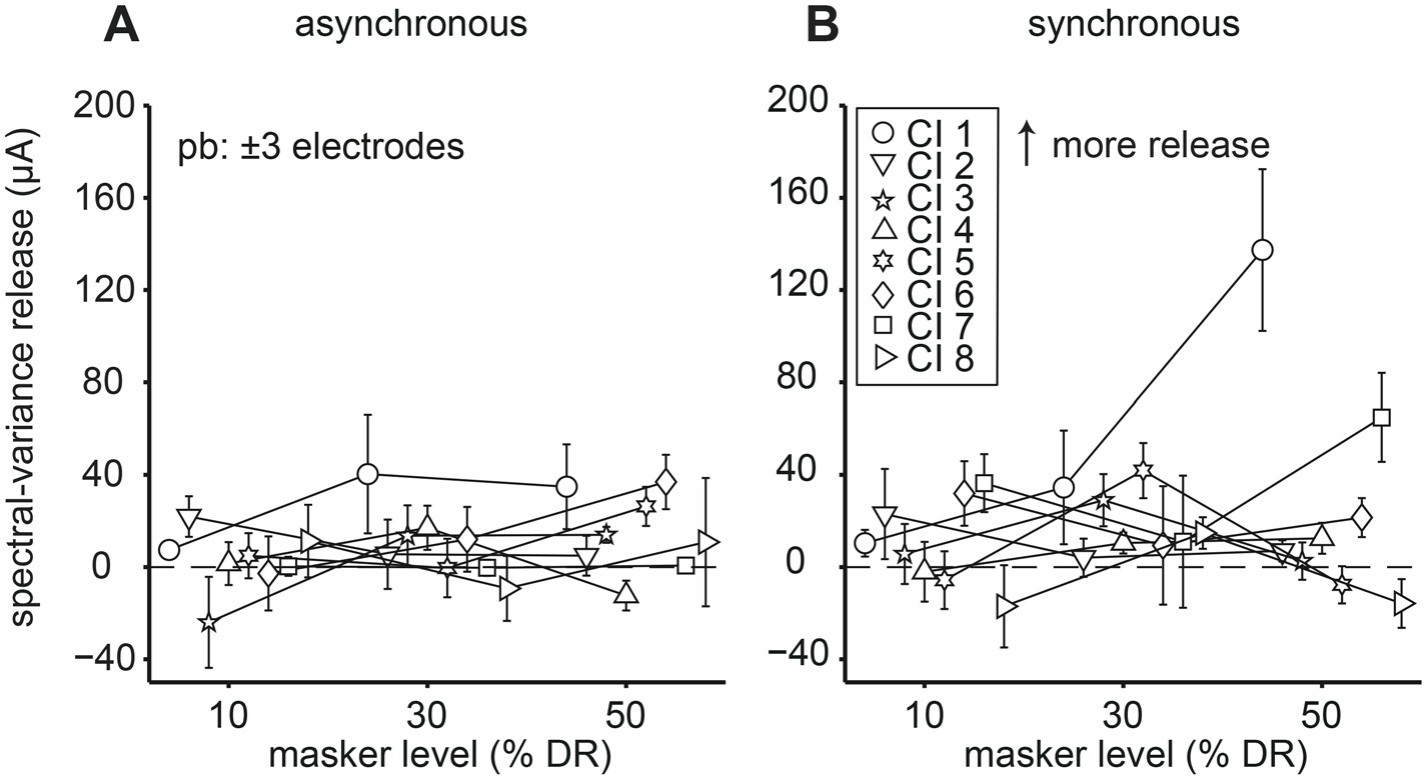
Effect of masker level on spectral-variance release. Data from all masker levels with a protected band (pb) of ±3 electrodes are plotted. (A) Asynchronous conditions. (B) Synchronous conditions. The amount of spectral-variance release equals the difference between median central (C-R) thresholds. The dashed line at zero demarcates no difference in central thresholds as a function of masker level (percentage of dynamic range, % DR). Different symbols represent individual listeners. Error bars indicate the standard deviation obtained with a bootstrap method (see main text). Higher values indicate more spectral-variance release.

We further analyzed performance by calculating the difference between thresholds obtained with the C and R maskers as a measure of spectral-variance release. These differences were plotted as a function of masker level for the asynchronous (Fig 5A) and synchronous (Fig 5B) conditions with higher values signifying more release. The error bars indicate the bootstrapped standard deviation (see Methods I). Most listeners, except for listeners CI 1 and CI 7, showed negligible amounts (close to 0 μA) of spectral-variance release independent of masker level and temporal condition. Note, however, that some listeners exhibited a peaked function (e.g. asynchronous: CI 4 and synchronous: CI 3 and CI 5) indicating modest amounts of spectral-variance release at the 30% masker level. In contrast, listener CI 1 exhibited a large increase in spectral-variance release across masker levels. In the asynchronous condition, spectral-variance release for listener CI 6 increased linearly with masker level and reached a maximum of ~40 μA. Spectral-variance release in listeners CI 1 and CI 3 increased between 10% to 30% DR masker level and plateaued at 50% DR masker level. In the synchronous condition, spectral-variance release for that listener reached ~160 μA. Note that the bootstrapped standard deviations could be quite large with increasing masker level (e.g., 30% DR: CI 1, 6, 7; 50% DR: CI 1, 7) (Fig 5B). This variability might be related to task difficulty [37]. The difference in spectral-variance release between the two temporal conditions is most likely due to ceiling and floor effects. That is, in the asynchronous conditions the listener performed close to ceiling so that addition of spectral-variance only slightly decreased detection thresholds. In the synchronous conditions, however, the listener operated at floor performance so additional cues could strongly decrease the signal detection threshold [27].

#### Central versus peripheral masking

There was no significant difference between C and P thresholds, irrespective of the temporal condition and masker level (Fig 3) (*p* > 0.05; Friedman’s test with Bonferroni correction for multiple comparisons). Fig 4C displays thresholds in the same scatter plot format as used previously, with individual absolute thresholds from C conditions plotted as a function of the corresponding P conditions. A linear fit to the data showed a small offset of ~16 μA (*R* ^2^ = 0.89; *p <* 10^−5^). Sixty-two percent of the points fell above the unity line indicating a small tendency for C thresholds to be higher than P thresholds but this difference was not significant (Kolmogorov-Smirnov test, *p* = 0.57). In contrast, listeners CI 6 and CI 7 showed higher P than C thresholds (<40% of points above the unity line).

#### Absolute loudness is not a reliable cue for masking release

We wondered if threshold differences between the three different masker types (P, C, and R) could be due to differences in loudness of these maskers. Accordingly, we performed a subjective 2-interval loudness comparison experiment with our CI listeners. We tested pairs of maskers, e.g. P vs. C masker, etc., and asked the listeners to indicate which of the two intervals contained the louder sound. We did not find any systematic differences in their loudness judgments of the maskers. Therefore we excluded loudness differences as a major contributing factor to the observed detection thresholds (data not shown).

#### Increasing protected bandwidth fails to improve spectral-variance release

**Fig 6:**
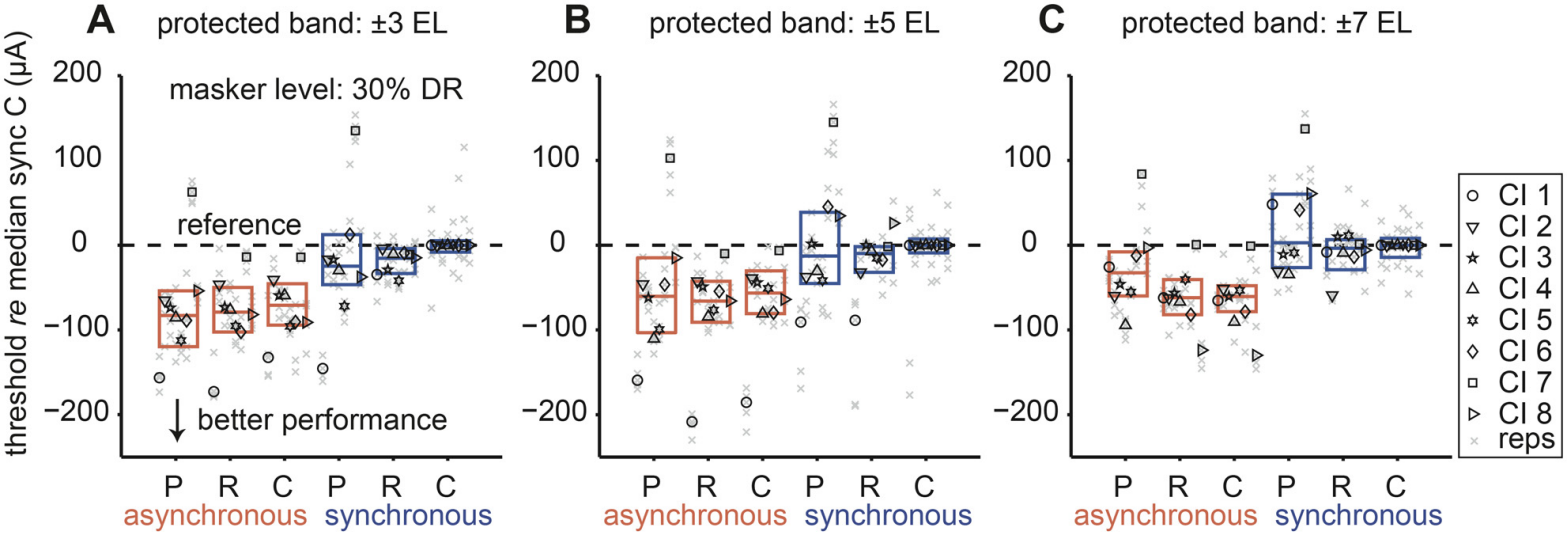
Effect of increased protected band on signal detection thresholds. Capital letters on abscissa correspond to peripheral (P) and central (C, R) maskers in both timing conditions. Signal detection thresholds (*re* each CI listener’s median, synchronous C threshold) are plotted as a function of stimulus. Three protected band conditions were tested at a masker level of 30% dynamic range (DR): (A) ±3, (B) ±5 and (C) ±7 electrodes (EL). All other conventions are as in Fig 3.

With a protected band of ±3 electrodes two CI listeners showed clear spectral-variance release from central masking (Fig 5B, CI 1, 7). We wondered whether increasing the protected band might enhance spectral-variance release. Fig 6 shows the effect of increased protected band from ±3 (re-plotted from Fig 3B) to ±5 and ±7 electrodes (Fig 6A, 6B, and 6C, respectively) at the same fixed 30% masker level. In general, the threshold difference between the two timing conditions persisted irrespective of the protected bandwidth (Friedman test, *p* < 0.05 for all comparisons at the group level). Peripheral thresholds were not significantly different from either central C or R thresholds (Friedman test, *p* > 0.05 for all comparisons). At ±7 protected band, P thresholds tended to be higher than both central C and R thresholds. Interestingly, four listeners (CI 1, 6, 7, 8) had a higher P detection thresholds compared to C and R thresholds in both temporal conditions, respectively (Fig 6C). In listener CI 7, increasing the protected band to ±5 and ±7 significantly increased differences between asynchronous P and C thresholds (Friedman test, *p* < 0.05).

**Fig 7:**
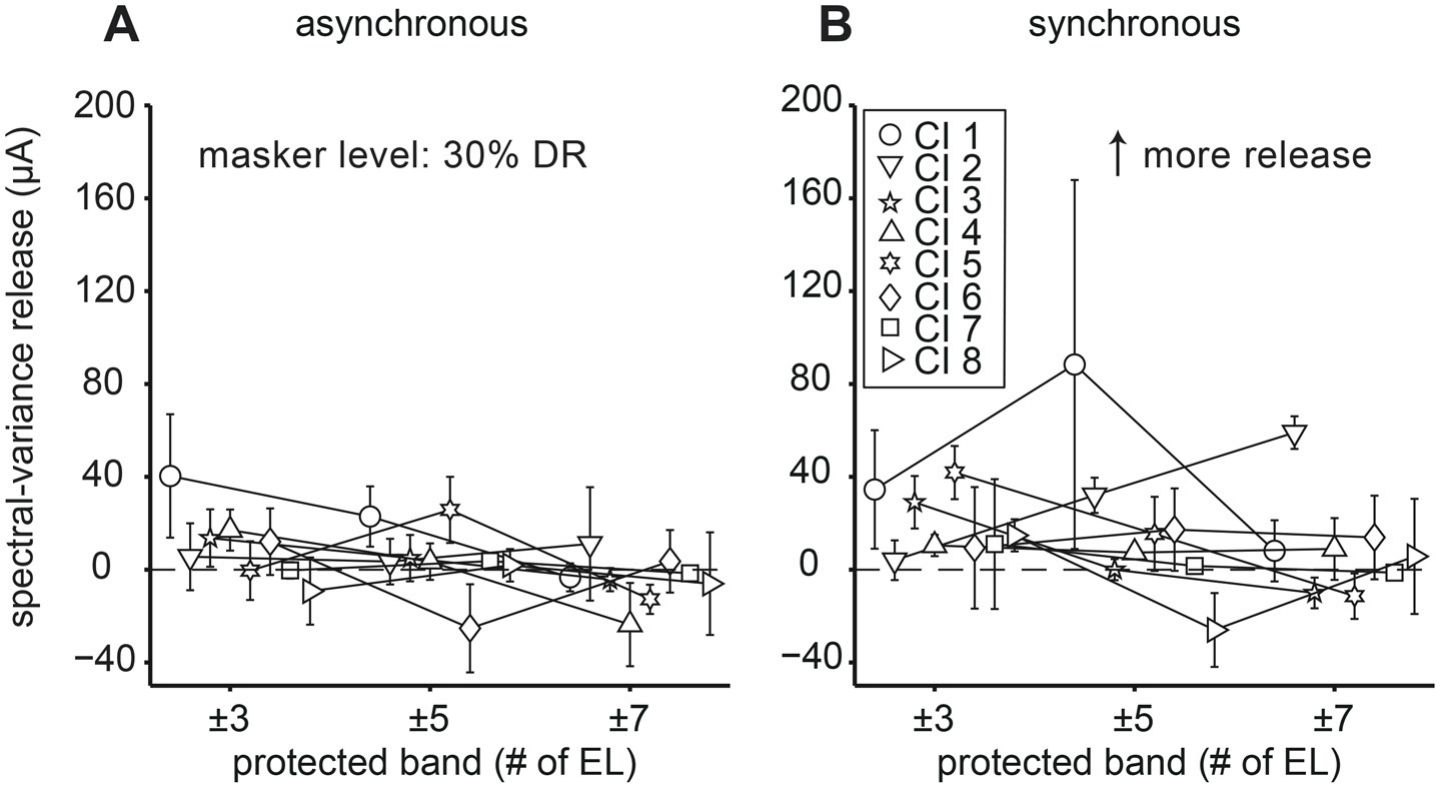
Effect of protected bandwidth on spectral-variance release. Data from all three protected band conditions (±3, ±5 and ±7 electrodes) at 30% dynamic range (DR) masker level are plotted. (A) Asynchronous conditions. (B) Synchronous conditions. The dashed line at zero demarcates no difference in central (C-R) thresholds as a function of protect band expressed in terms of number of electrodes (# of EL). All other conventions are as in Fig 5.

The amount of spectral-variance release from central masking as a function of protected bandwidth is shown in Fig 7. The error bars indicate the bootstrapped standard deviation (see Methods I). In the asynchronous conditions (Fig 7A) spectral-variance release either stayed constant (CI 2, 3, 7, 8), decreased slightly (CI 1, 4), peaked (CI 5) or decreased (CI 6) at ±5 protected bandwidth (CI 5). We saw similar trends in the synchronous conditions (Fig 7B) in that spectral-variance release could stay constant (CI 4, 6, 7), decrease (CI 3, 5), increase (CI 2), peak (CI 1) or decrease (CI 8) at a protected bandwidth of ±5 (CI 1). The overall trend in the asynchronous conditions was decreasing spectral-variance release with increasing bandwidth. An increase in protected bandwidth might have had two effects: 1) reduced the amount of peripheral masking thereby decreased thresholds in the presence of central maskers, and 2) increased similarity between the central C and R maskers (due to the restricted number of unique stimulating electrodes), which in turn would lead to similar detection thresholds in the two masker conditions. These two counteracting effects might explain the general trend seen in the asynchronous conditions.

The observed pattern in the synchronous conditions was more complex and might reflect the ambiguity of the stimuli, in that here temporal cues hampered and spectral-variance cues facilitated signal detection. Listeners CI 3, 4, 5, 6, 7, and 8 did not benefit from increased protected bandwidth. Listeners CI 1 and CI 2 benefited from increased protected bandwidth. While Listener CI 2 exhibited a linear increase of spectral variance release, listener CI 1 showed non-linear release. Interestingly, this listener had relatively narrow peripheral filters (Fig 1C, dark blue). The non-linear, peaked function might reflect an optimum for the two counteracting effects of increased protected bandwidth discussed above. Note also that with the ±5-electrode wide protected band variability was largest, again indicating increased difficulty of the task and the possible involvement of central auditory processing.

#### No correlation between spectral-variance release and audiological factors

We performed correlation analyses between spectral-variance release with audiological factors including age, age of hear loss onset, deafness duration, years of hearing aid usage and years of CI usage, respectively, and found no correlations. Furthermore, correlation analysis between spectral-variance release and speech recognition with +10 dB signal to noise ratio (Hearing In Noise Test (HINT)) in our sample of eight CI listeners revealed no correlation; a much larger sample size, however, would be necessary to conclude with certainty whether our measures of spectral-variance release correlate with speech-in-noise recognition scores.

## Experiment II: Acoustic Simulation of CI Peripheral and Central Masking

### Rationale

We designed Experiment II to test the hypothesis that broad peripheral filters could degrade the spectral-variance cues, while leaving the temporal cues intact; thus offering a potential explanation for the results observed in CI listeners (Experiment I). We used noise bands to acoustically simulate CI listening to test this hypothesis in five NH listeners. Simulated peripheral filter width was controlled by adjusting the noise bandwidth. We predicted that NH listeners would show elevated R thresholds *re* C thresholds with increasing overlap between noise bands, which would mirror generally reduced spectral-variance release in CI listeners.

### Experiment II. Methods

#### Normal hearing listeners

Five NH listeners (three females; age 24–72, mean age 44) were recruited to participate in the simulation experiments. All listeners had normal audiograms with pure tone thresholds below 20 dB HL at low frequencies with the exception of listener NH 3 (age 63) and NH 4 (age 72) whose thresholds were 35 dB HL and 45 dB HL at 8 kHz, respectively and 70 dB HL at 12 kHz, for both listeners.

#### Acoustic stimuli

We simulated implant listening in NH listeners using 22 noise bands (representing 22 frequency channels) of varying bandwidth centered at logarithmically-spaced frequencies ranging from 0.2–14 kHz in steps of 0.3 octaves. Instead of specifying electrode numbers, burst durations and timings for the HEINRI system, acoustic stimuli were generated in MATLAB, amplified via a sound card (Creative Labs E-MU 0404 USB digital audio system, Creative Technology Ltd., Singapore, 16-bit, 44.1 kHz) and presented monoaurally via calibrated circumaural headphones (HDA-200, Sennheiser electronic GmbH & Co. KG, Wedemark, Germany). To simulate non-overlapping and overlapping CI filters, we tested three noise bandwidth conditions expressed in octaves. Note that we did not correct for edge effects, i.e., we clipped the noise bands at 0.2 and 14 kHz. Therefore, noise bands with high and low center frequencies are asymmetrical and as a result can have bandwidths spanning at most 1.0 octave. The signal was a noise band with a center frequency of 1851 Hz and a protected band corresponding to ±3 center frequencies or 2.0 octaves was used in all three conditions. Both signal and masker were 40-ms noise bursts, pulsed four times at a rate of 5/s. The same six stimulus conditions used for the electric stimuli were used for acoustic stimuli.

#### 2AFC paradigm for testing NH listeners

To test the NH listeners we used the 2AFC paradigm, stimulus generation routines, and software similar to the ones used in Experiment I. We presented individual masker bands at 60 dB SPL and initially set the signal level to 70 dB SPL. On subsequent repetitions, we set the signal level to 10 dB SPL above the previously tracked threshold level. Large step sizes of 3 dB were used for the first three reversals and small step sizes of 1 dB were used for the next seven reversals. We repeated this measurement for each noise bandwidth condition. Detection thresholds were based on the median of five repetitions in each NH listener except for listener NH 4 (three repetitions). We used the same masker presentation scheme as in the electric experiments with the three overlap conditions presented in random order. Before detecting masked signals, listeners tracked an unmasked threshold for the signal in each overlap condition to provide a baseline against which to compare masked thresholds.

#### Training

We started training of signal band detection in the presence of asynchronous R maskers with no overlap. Initially, we set the masker level to 40 dB SPL to facilitate detection. After obtaining a threshold at this masker level, training was repeated at increased masker levels (50 and 60 dB SPL) until the threshold tracking curves stabilized (i.e., plateaued after ten reversals).

### Experiment II. Results

#### Simulated wide peripheral filters degrade spectral-variance cues while leaving temporal cues intact

**Fig 8:**
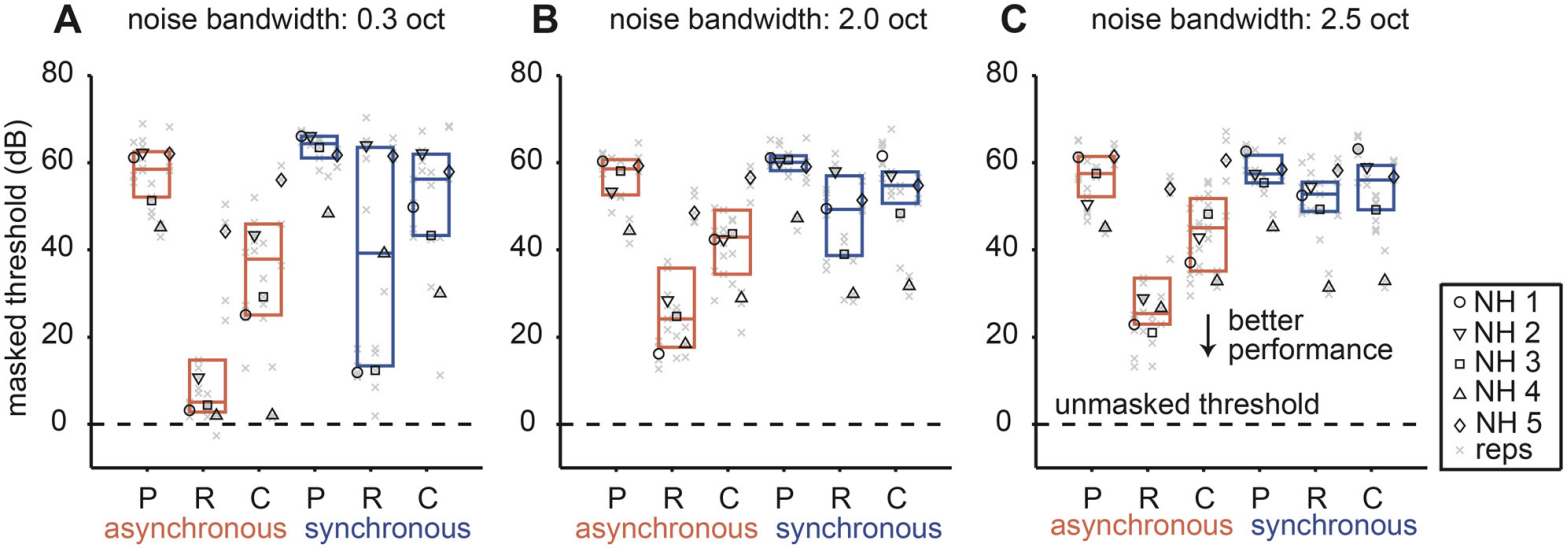
Effect of noise bandwidth on signal detection thresholds in CI simulation. Capital letters on abscissa correspond to peripheral (P) and central (C, R) maskers in both asynchronous (red) and synchronous (blue) timing conditions. Detection thresholds *re* unmasked threshold for the signal presented in quiet (dashed line) is plotted as a function of stimulus. Three different noise bandwidth conditions are shown: (A) ±0.3, (B) ±2.0 and (C) ±2.5 octaves. All other conventions are as in Fig 3.

Fig 8 shows the effect of noise bandwidth on signal detection thresholds from the simulations in a similar format as used in Fig 3. Detection thresholds were referenced to those measured in an unmasked, signal-only condition (dashed line). For a noise bandwidth of 0.3 octaves (Fig 8A), thresholds largely mirrored our previous results with multi-tone maskers [27]. Detection thresholds were lower in the asynchronous (red) *re* synchronous (blue) conditions, which demonstrated that central processing of the temporal cues remained intact. Central R maskers tended to yield lower thresholds *re* central C maskers. In contrast to some CI listeners, thresholds for NH listeners in the presence of peripheral maskers were higher than in the corresponding central masker conditions. Also note that as previously reported in the informational masking literature [22] inter-listener variability was large. For example, listener NH 4 experienced little central masking in the asynchronous conditions (Fig 8A, 8C and 8R). Only listeners NH 1 and NH 3 experienced central masking release in the synchronous condition (Fig 8A). On a group level, as noise bandwidth increased: 1) detection thresholds in the presence of central, but not peripheral, maskers increased and 2) inter-listener variability decreased (compare Fig 8A, 8B and 8C).

The P thresholds were significantly higher than the R thresholds (*p* < 0.05; Friedman’s test using a Bonferroni correction) and the C thresholds (*p* < 0.05; Friedman’s test using a Bonferroni correction) (Fig 8). In the synchronous conditions, only R thresholds (*p* < 0.05; Friedman’s test using a Bonferroni correction) were significantly higher than the thresholds obtained with P maskers. Note that with increased noise bandwidth the difference between thresholds obtained with R and C maskers decreased notably.

**Fig 9:**
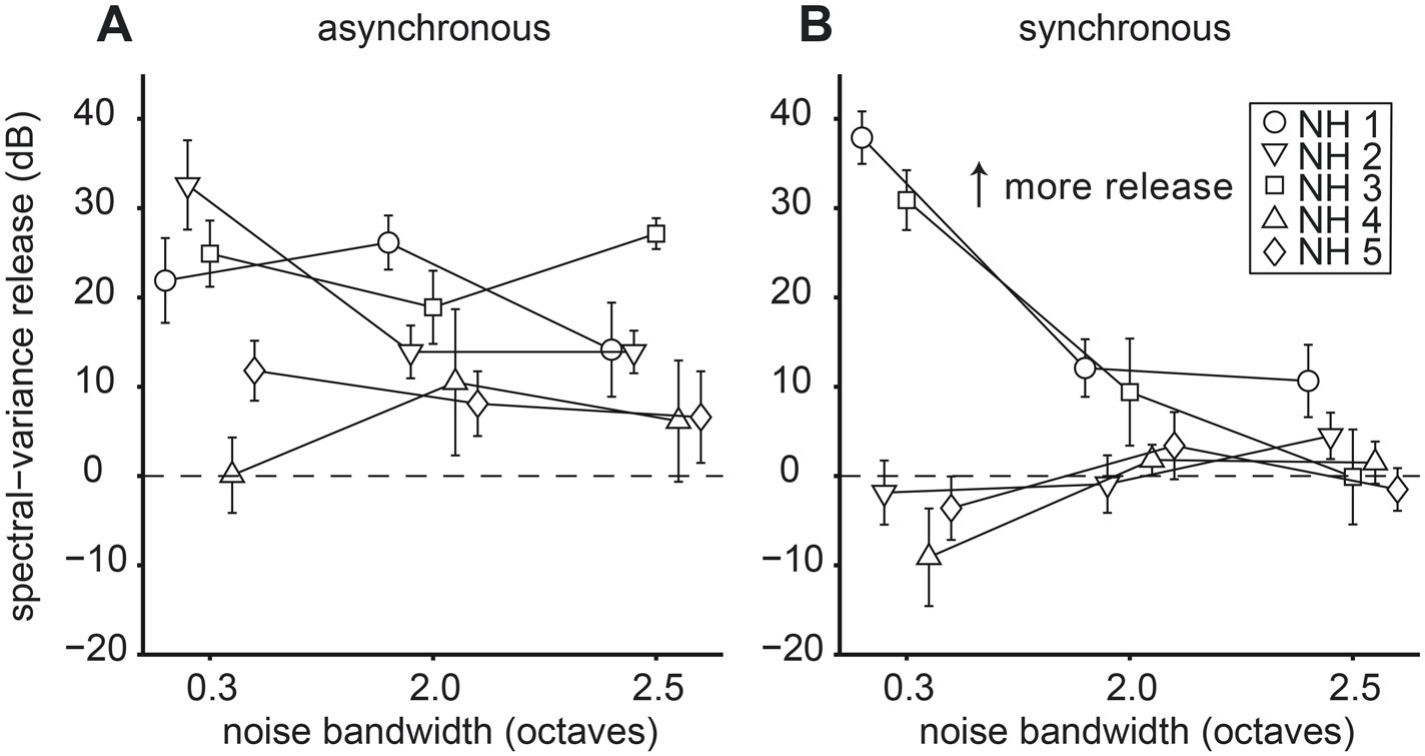
Effect of noise bandwidth on spectral-variance release. (A) Asynchronous conditions. (B) Synchronous conditions. The dashed line at zero demarcates no difference in central (C-R) thresholds as a function of noise bandwidth expressed in octaves. All conventions are as in Fig 5.

To quantify the effect of noise bandwidth on spectral-variance release we plotted C-R threshold differences and bootstrapped standard deviations (see Methods I) in the asynchronous (Fig 9A) and synchronous (Fig 9B) conditions. In general, spectral-variance release either stayed constant or decreased across the three bandwidth conditions while intra-subject variability was relative constant across conditions (~10 dB). Individual thresholds, however, could vary considerably with some listeners exhibiting no spectral-variance release and others exhibiting large amounts of release. This observation is in accordance with previous reports and is commonly attributed to different listening strategies, i.e. inter-listener differences in central processing [25,26]. It is likely that prior to implantation individual CI listeners also would have employed different central listening strategies. This effect might have influenced detection thresholds in our CI listeners in addition to any effects due to electrical stimulation.

In NH listeners who benefited from the spectral-variance cues, an increase in noise bandwidth decreased the amount of spectral-variance release, e.g. listener NH 3 showed a decrease of ~40 dB from 0.3 to 2.5 octave bandwidth (Fig 9B). This seemed to suggest that deteriorated peripheral input, simulated here by increasing overlapping spectra, severely hampered central processing. In relation to CI listeners, the NH data seemed to indicate that wide peripheral filters were one aspect of peripheral encoding that could contribute to the reduced spectral-variance release observed across CI listeners. These acoustic simulations of implant listening seemed to suggest that restricted frequency resolution in the periphery weakened spectral-variance cues but not temporal cues accessible to the central auditory system.

## Discussion

Our findings supported the hypothesis that post-lingually deaf CI listeners retain certain central processing abilities, but these are severely impaired by poor peripheral encoding. We showed that: 1) central processing of timing remained intact in all CI listeners, whereas central processing of spectral-variance seemed to be maintained in only two out of eight CI listeners, and 2) simulating implant listening in NH listeners with normal central processing showed that broad peripheral filters limited the amount of spectral-variance release from central masking.

### CI listeners can retain NH-like central auditory processing

All CI listeners could use the temporal cues for signal detection, but not all CI listeners fully benefitted from the spectral-variance cues (Figs 4B and 5). In particular, listeners CI 1 and CI 7 showed the largest spectral-variance release from central masking. One factor potentially influencing spectral-variance release could have been filter bandwidth. In an attempt to improve spectral resolution, we tested increased protected band conditions (Figs 6 and 7). In the asynchronous conditions, spectral-variance release tended to decrease with increasing protected bandwidth. In the synchronous conditions, however, the observed spectral-variance release pattern was more complex. Increasing the protected band on the one hand further limited the potential for peripheral masking of the signal electrode, but on the other hand decreased the number of electrodes available for the central masking stimuli, i.e. the R masker became more similar to the C masker. Therefore one might conjecture that listeners CI 1 and CI 7 have more perceptually independent channels in comparison to the other CI listeners in our sample, which enabled better use of spectral-variance cues to segregate the masked signal. Although the results did not reach significance in our small sample of CI listeners, the trend in Fig 5 suggested that listeners CI 1 and CI 7 could make use of the spectral-variance cues to gain release from central masking. Central processing of spectral variance, however, is largely hampered by poor peripheral encoding.

Conversely, spectral resolution as estimated by peripheral filter width does not fully account for central masking release. Listener CI 4, for instance, had extremely narrow filters but did not benefit from spectral-variance cues (Fig 1C, green). Conversely, listener CI 1 had relatively narrow filters but showed considerable spectral-variance release (Fig 1C, red). We, therefore, considered the possibility that other peripheral factors, such as the uniformity and health of surviving auditory neurons and their proximity to the CI electrode [14] are potential sources of degraded peripheral input which could in turn limit central masking release. By extension, poor peripheral encoding of spectral information hampers central processing of speech. Reduced spectral resolution due to a limited number of perceptual spectral channels and/or channel interactions across electrodes could be responsible for the absence of fine spectro-temporal cues. In turn loss or degradation of these cues may contribute to poor speech understanding in noise, especially in dynamically changing backgrounds in which there are competing speakers or modulated noise [13]. Degraded temporal fine structure processing in CI subjects has proven to be detrimental for speech understanding in noise [38].

Evidence from human intra-cranial electrocorticography concur with our behavioral results that CI listeners retain NH-like central auditory processing [39]. Intra-cranial recordings of responses to CI stimulation in a human bilateral CI patient revealed cortical responses seemed quite similar to those obtained in NH, epilepsy patients [39]. Latencies of the auditory evoked potential waveform peaks (P_α_, N_α_, P_β_, N_β_) in response to 100-Hz clicks in the CI subject were all within the range of latencies seen in 10 NH control participants. Considering how spectral resolution from independent stimulation channels provided by the implant is limited [14], CI listeners depend heavily on temporal envelope information for speech perception.

### Simulated CI listening highlights individual differences in central auditory processing

We initially hypothesized that broad peripheral CI filters could fully account for degraded central processing of spectral variance. Indeed, less spectral-variance release with increasing noise bandwidth observed in NH listeners (Experiment II) seemed to support this view. Modeling broad peripheral filters acoustically with noise bands of varying bandwidth, however, could not account for *all* differences observed between acoustic and electric hearing. NH listeners still performed better with ‘wider-band’ stimuli (i.e., showed spectral-variance release) in comparison to CI listeners with wide peripheral filters. Thus, relative differences in the degree of spectral-variance release suggest differences in central processing abilities and the neuronal representation of relevant cues between the two listener groups. The limited dynamic range in CI listeners might reduce spectral contrast [40]. We did not attempt to simulate this factor in the present study. The influence of a limited dynamic range could, for example, be simulated by using a limited number of quantized loudness steps across the full acoustic dynamic range or by adding additional broadband noises to compress the stimuli into a limited acoustic dynamic range.

Inter-listener variability, generally, tended to be large with central (informational) masking stimuli, i.e. across NH listeners these complex maskers could either strongly elevate or only mildly raise detection thresholds [25]. By design, our stimuli contained cues that could either facilitate or hamper signal detection. For example, in the easiest masking condition, asynchronous R, both the temporal and the spectral-variance cues could facilitate signal detection. In this case listeners could potentially reach ceiling performance. Conversely, in the most difficult case (synchronous C) performance could have been close to floor performance. In the extreme cases (floor and ceiling performance), response variability would be small. Conditions in between these two extremes, however, would have led to increased response variability if the listener were capable of accessing all or some of the cues. In contrast to the other CI listeners, listener CI 1 exhibited not only spectral-variance release but also a systematic increase in response variability. Accordingly, we surmise that under the most favorable peripheral encoding conditions, i.e. relative narrow peripheral filters and large dynamic range as seen in listener CI 1, CI listeners retain central processing abilities similar to those of NH listeners. Currently, the origin of inter-listener differences both in CI and NH listeners remains unclear.

### Implications for speech perception

Limited spectral resolution and dynamic range distort the internal representation of spectral contrast important for segregating speech from noise [40,41]. Friesen and colleagues [42] tested recognition of simple sentence material presented at a 5 dB signal-to-noise ratio, and showed that more spectral channels were required in noise compared to in a quiet condition to achieve similar performance. They also demonstrated that most CI listeners are not able to fully utilize the spectral information provided by the number of electrodes used in their implant. Their results align with our findings. Thus, for improving speech-in-noise perception, it seems vital to increase frequency selectivity by e.g. developing new types of auditory prostheses with improved spectral resolution [43] and to increase the dynamic range of CIs by e.g. developing better electro-neural interfaces for current generation implants [44,45]. It is also important to realize that the central auditory system of CI listeners still employs central processing strategies despite the artificial nature of electrical stimulation. With narrow peripheral filters and a large dynamic range, CI listeners might be able to exploit not only temporal cues but also better perceive spectral-variance cues, which are important factors in speech understanding in complex auditory scenes.
